# The Predictive Role of Intraoperative Blood Transfusion Components in the Prognosis of Heart Transplantation

**DOI:** 10.3389/fcvm.2022.874133

**Published:** 2022-05-20

**Authors:** Yidan Zheng, Li Xu, Ziwen Cai, Jingrong Tu, Yuqi Liu, Yixuan Wang, Si Chen, Nianguo Dong, Fei Li

**Affiliations:** ^1^Department of Cardiovascular Surgery, Union Hospital, Tongji Medical College, Huazhong University of Science and Technology, Wuhan, China; ^2^Key Laboratory of Organ Transplantation, Ministry of Education, NHC Key Laboratory of Organ Transplantation, Key Laboratory of Organ Transplantation, Chinese Academy of Medical Sciences, Wuhan, China

**Keywords:** heart transplantation, blood transfusion, red blood cell, platelet, plasma

## Abstract

**Purpose:**

To evaluate the influence of transfusion amount of blood components on the prognosis of patients after heart transplantation (HTx).

**Methods:**

From 1 January 2015 to 31 December 2020, 568 patients underwent HTx in our institute. A total of 416 recipients with complete datasets were enrolled in the study for final statistical analysis according to the inclusion criteria. The optimal cut-off values for intraoperative transfusion of red blood cell (RBC), platelet, and plasma were determined with receiver operating curve analysis. Univariate and multivariate Cox regression analyses were applied to compare baseline data of patients divided by the transfusion amounts of RBC, platelet, and plasma. Propensity score matching was used to enable the direct comparison of outcomes.

**Results:**

The Kaplan–Meier analysis revealed that transfusion amounts of RBC and plasma were independently associated with overall mortality, increased intensive care unit stay time, and major adverse events after transplantation. The multivariate Cox regression analysis suggested that neurological complications (*p* = 0.001), liver damage (*p* = 0.011), and respiratory complications (*p* = 0.044) were independent risk factors for overall mortality after HTx. Combining indicators presented a good predicting effect of peritransplant period mortality (AUC = 0.718).

**Conclusion:**

The mortality of HTx was significantly related to the high-amount transfusion of RBC and plasma. Comprehensively considering the components of blood transfusion obtained better predictive results of peritransplant period survival than solely considering a single component.

## Introduction

Cardiovascular diseases are the leading cause behind the mortality of population in modern industrialized nations. To date, heart transplantation (HTx) remains the golden standard therapeutic option for end-stage heart diseases. A report from the American Heart Association revealed that between 1987 and 2012, 40,253 people who waited for HTx had a median survival of 2.3 years, while 26,943 who received transplants gained median survival of 9.5 years, 5.0 life-years saved per patient (1). Due to the shortage of donor grafts and effective organ-preserving techniques, only about 6,000 patients have the chance to undertake HTx worldwide per year (2). Accordingly, it is of great significance to thoroughly recognize the clinical characteristics of recipients, and explore and intervene about the risk factors which would contribute to predicting the mortality potential of patients receiving HTx.

Blood transfusion is a well-established therapy widely applied in cardiac surgery. Evidence suggested that although blood transfusion tended to bring patients benefits during the operation, risks and costs were also associated. Several studies have separately discussed the association between the outcome of cardiac surgery and blood transfusion components, such as platelet and plasma (3, 4). However, the impact of the blood transfusion on the prognosis of HTx is still lacking.

In this study, we comprehensively evaluated the correlation between the transfusion amount of three major blood components and the clinical outcome of HTx. Based on statistical analysis, we further investigated the prognostic value of red blood cells (RBCs), platelet, and plasma transfusion in HTx.

## Methods

### Study Population

This retrospective study reviewed the data from patients undergoing orthotopic HTx in our center from 1 January 2015 to 31 December 2020. Patients under 18 years of age and recipients with retransplant, multiple organ transplants, or umpteen data missing were excluded from the study. Our institution applied an electronic medical records system, from which baseline biochemical characteristics and clinical measures were conducted.

### Ethical Statement

The ethics approval was obtained from the Ethics Committee of Tongji Medical College of Huazhong University of Science and Technology (IORG No: IORG0003571). All donor grafts were obtained from donation after brain death and assigned by the China Organ Transplant Response System according to Chinese laws of deceased organ donation. The study protocol followed was under the guidance of the national protocol for China category I and conformed to the Declarations of Helsinki and Istanbul.

### Follow-Up Data and Outcome

The clinical follow-up information of recipients was collected from regular clinical visits, telephone interviews, or the Internet. Patients were followed up until 31 December 2020. Overall survival was defined as the absence of overall mortality. Peritransplant period (30 days after HTx) mortality and 1-year mortality were also collected in this study. The mortality data were retrieved from the China Heart Transplant Registration Network, where all deaths are registered under the requirement of law.

### Data Collection and Subgroup Division

Baseline characteristics contained the preoperative variables, personal history, and donor variables. Pre-transplant variables are selected based on whether it is a risk factor or it is a potential risk factor, and the variables that describe the physical conditions of patients are also included for sure. The preoperative variables consisted of demographic data of recipients and donors, preoperative laboratory indicators, and preoperative treatments. The demographic data included gender, age, body mass index, and blood type. The preoperative laboratory indicators included RBC count, levels of blood platelet, white blood cell count, hemoglobin (Hb), albumin, serum creatinine (Cr), low-density lipoprotein, and triglyceride. The preoperative treatments included intra-aortic balloon pump (IABP) and extracorporeal membrane oxygenation (ECMO). Personal history included the history of smoking, cardiac surgery, hypertension, and diabetes. Charlson Comorbidity Index (CCI) was also consisted to assess the status of comorbidity, generated from selected chronic diseases.

Postoperative events data were also included. Early postoperative events included postoperative use of Continuous Renal Replacement Therapy (CRRT), IABP, and ECMO, respiratory, neurological, and renal complications, liver damage, septic shock, secondary thoracotomy, and ICU stay time. Mortality variables contained a peritransplant period (defined as 30 days after HTx), 1-year and 5-year all-cause mortality.

In addition to the routine indicators, the amounts of transfusion of RBC, platelet, and plasma were also measured. Receiver Operating Characteristic (ROC) analysis was performed and the optimal cut-off values were determined by the max Youden's index on the curve. We also calculated the area under the ROC curve (AUC) to confirm the distinctive role of RBC, platelet, or plasma transfusion in overall mortality. The study population was divided into high-amount groups and low-amount groups via the classification criteria of RBC, platelet, and plasma, respectively.

### Statistical Analysis

SPSS 26.0 (IBM Corp., Armonk, NY, USA) and R version 4.0.1 with the packages Survminer, survival, optimal Cutpoints, ggplot2, dplyr, and table one were used for statistical analysis. Categorical variables were presented as the number of recipients (percentage). The continuous variables were presented as mean ± standard deviation (SD) for variables following a normal (Gaussian) distribution and median [interquartile range (IQR)] for most variables that did not follow the normal distribution. The comparisons among groups were performed by Chi-square test or Fisher's precision probability test for continuous variables, and Mann–Whitney U rank-sum test for categorical variables, as appropriate. Propensity score matching (PSM) was applied to adjust the unbalanced covariates from baseline characteristics. The univariable survival analysis and the difference between RBC, platelet, and plasma groups were statistically evaluated by the Kaplan–Meier method and examined by the log-rank test. Hazard ratios (HR) and corresponding 95% confidence intervals (CI) were estimated by the univariate and multivariate Cox proportional hazards regression model. A two-tailed *p* < 0.05 was defined as statistical significance.

## Results

**Figure 1:**
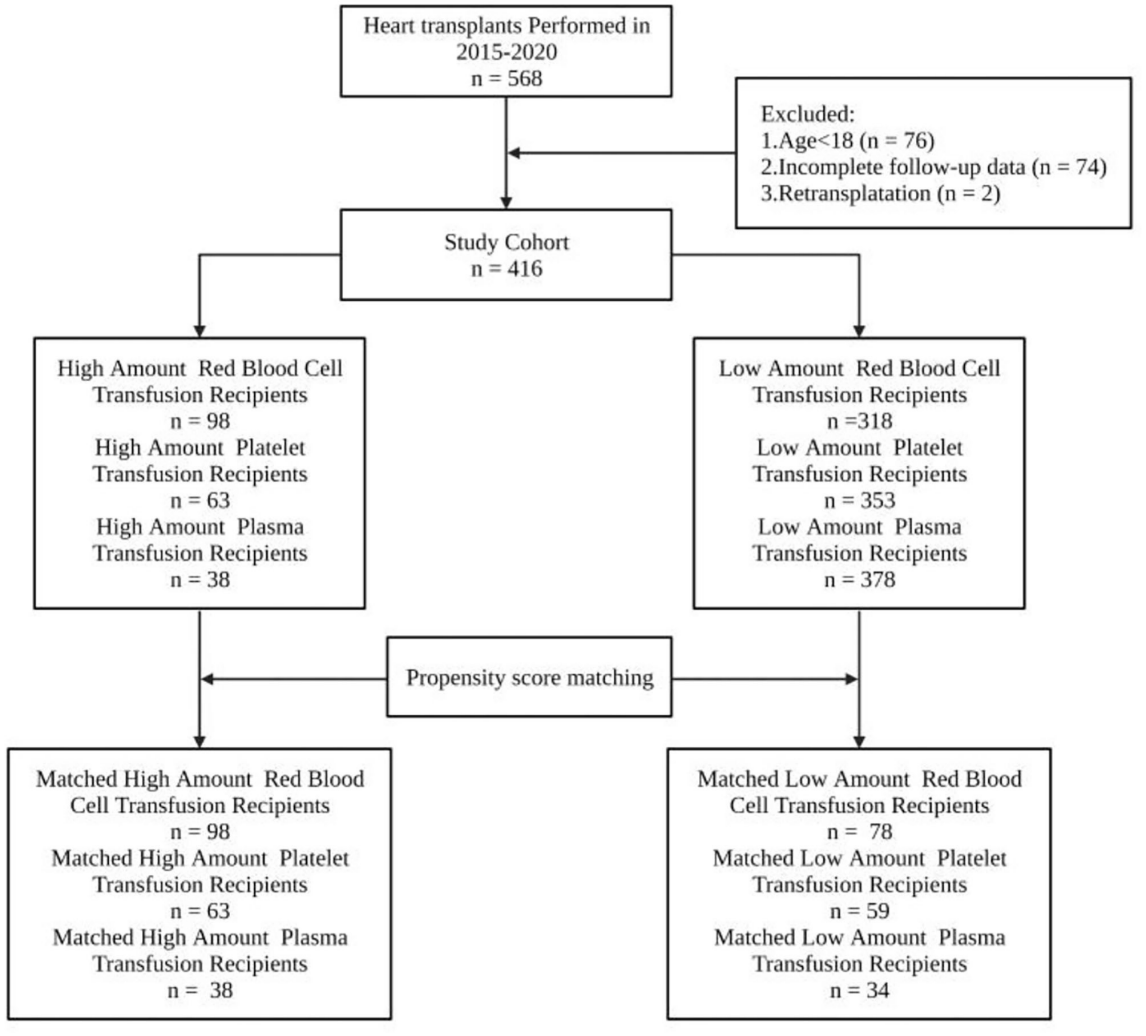
All 568 cardiac transplant patients at Wuhan Union Hospital from January 2015 to December 2020 were recruited. Patients with ages under 18 years, umpteen incomplete follow-up and transfusion data, or receiving retransplant were excluded from the study. Among the 568 recipients, 76 were under the age of 18, 74 had umpteen incomplete follow-up data, and 2 received retransplant. About 416 recipients were finally enrolled in the study cohort according to the inclusion criteria.

A total of 568 patients received HTx in our center during the study period. Among the 568 recipients, 76 were under the age of 18, 74 had umpteen incomplete follow-up data, and 2 received retransplant. Four hundred sixteen recipients were finally enrolled in the study cohort according to the inclusion criteria (Figure 1).

### The Optimal Cut-Off Values of the Transfusion of RBC, Platelet, and Plasma

ROC analysis was performed to verify the optimal transfusion cut-off values of three blood components to predict the survival outcome. Results of the analysis showed that the areas under the curve (AUC) were 0.630 (*p* < 0.05, 95% CI 0.564–0.697), 0.582 (*p* < 0.05, 95% CI 0.514, 0.651), and 0.566 (*p* < 0.05, 95% CI 0.493–0.639) for RBC, platelet, and plasma, respectively. The optimal cut-off values were determined by the Youden index as nine units for RBC, three packages for platelet, and 1,250 ml for plasma.

**Figure 2:**
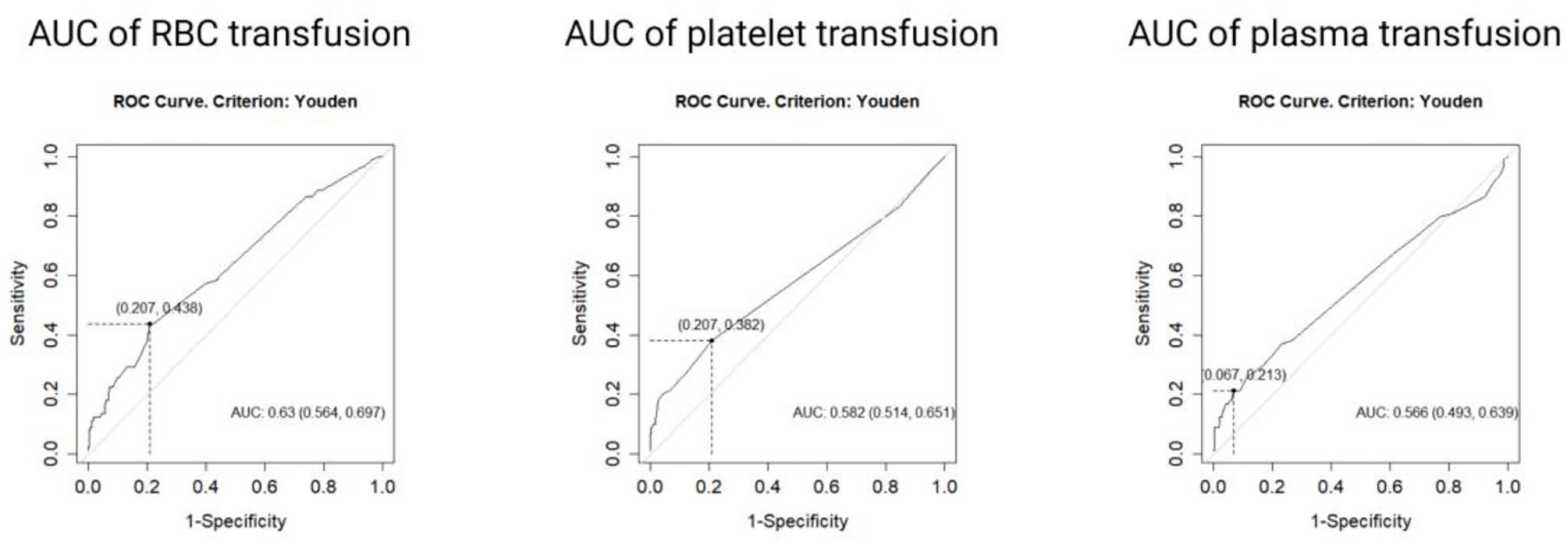
Receiver operating curves analysis was performed on the whole population. According to Youden index, the transfusion cut-off values of three blood components were 9 units for red blood cell (AUC = 0.63, 95%CI 0.564, 0.697), 2 packages for platelet (AUC = 0.582, 95% CI 0.514, 0.651), and 1,250 ml for plasma (AUC = 0.566, 95% CI 0.493, 0.639).

According to the cut-off values, the study population was divided into three pairs of groups: low RBC transfusion (≤ 9 units, *n* = 318) and high RBC transfusion groups (>9 units, *n* = 98); low platelet transfusion (≤ 3 packages, *n* = 353) and high platelet transfusion groups (>3 packages, *n* = 63); low plasma transfusion (≤ 1,250 ml, *n* = 378) and high plasma transfusion (>1,250 ml, *n* = 38), respectively (Figure 2).

### Baseline Characteristics and Intraoperative Blood Transfusion

Four hundred sixteen recipients who received HTx in Wuhan Union hospital from January 2015 to December 2020 were retrospectively introduced in this study, including 325 males and 91 females. Table 1 presented the baseline characteristics of recipients before and after propensity score matching. The relationship and differences of blood biomarkers, clinical characteristics, and personal history in low/high amount transfusion groups were analyzed. A high amount of RBC transfusion was significantly related to a low count of red blood cells (*p* < 0.001), low hemoglobin (*p* < 0.001), low albumin (*p* = 0.001), and high serum creatinine (*p* = 0.001). A high amount of platelet transfusion was significantly related to low red blood cell count (*p* = 0.037), low albumin (*p* = 0.001), and high serum creatinine (*p* = 0.009). A high amount of plasma transfusion was significantly related to a low count of RBC (*p* = 0.028), low albumin (*p* < 0.001), and low triglyceride (*p* = 0.046).

**Table 1:** Baseline characteristics and operative data of the 416 patients in the study cohorts.

**Variables**		**Before matched**				**After matched**			
	**level**	**Overall**	**Low (≤9 units)**	**High (>9 units)**	***p***	**Overall**	**Low ≤9 units**	**High (>9 units)**	***p***
**Red blood cell**									
*n*		416.000	318.000	98.000		185.000	87.000	98.000	
**Recipient preoperative variables**									
Gender (%)					0.048				0.825
	Male, *n*	325 (78.1)	256 (80.5)	69 (70.4)		128 (69.2)	59 (67.8)	69 (70.4)	
	Female, *n*	91 (21.9)	62 (19.5)	29 (29.6)		57 (30.8)	28 (32.2)	29 (29.6)	
Age, y		50.00 [39.00, 57.00]	50.00 [38.25, 56.00]	53.00 [40.00, 58.75]	0.185	52.00 [39.00, 59.00]	49.00 [38.50, 60.00]	53.00 [40.00, 58.75]	0.647
BMI, kg/m^2^		22.84 [19.90, 25.36]	23.20 [20.56, 25.88]	21.53 [18.92, 24.24]	0.001	21.45 [19.07, 24.03]	21.45 [19.24, 23.73]	21.53 [18.92, 24.24]	1.000
Blood type (%)					0.964				0.858
	A	138 (33.2)	106 (33.3)	32 (32.7)		64 (34.6)	32 (36.8)	32 (32.7)	
	B	116 (27.9)	88 (27.7)	28 (28.6)		50 (27.0)	22 (25.3)	28 (28.6)	
	O	132 (31.7)	100 (31.4)	32 (32.7)		58 (31.4)	26 (29.9)	32 (32.7)	
	AB	30 (7.2)	24 (7.5)	6 (6.1)		13 (7.0)	7 (8.0)	6 (6.1)	
Red blood cell count 10^∧^12/L		4.44 [4.00, 4.83]	4.50 [4.14, 4.89]	4.11 [3.61, 4.65]	<0.001	4.19 [3.78, 4.64]	4.25 [3.92, 4.61]	4.11 [3.61, 4.65]	0.138
Platelet count, 10^∧^9/L		171.00 [134.75, 218.25]	173.50 [136.25, 220.00]	166.50 [132.00, 211.50]	0.188	168.00 [134.00, 212.00]	170.00 [139.50, 215.50]	166.50 [132.00, 211.50]	0.551
White blood cell count, 10^∧^9/L		6.38 [4.95, 7.99]	6.36 [5.11, 7.88]	6.46 [4.49, 8.62]	0.731	6.43 [4.70, 8.25]	6.43 [5.12, 7.83]	6.46 [4.49, 8.62]	0.699
Hemoglobin, g/L		136.50 [119.75, 147.00]	138.00 [125.00, 149.00]	124.00 [110.25, 139.75]	<0.001	126.00 [113.00, 138.00]	127.00 [117.00, 138.00]	124.00 [110.25, 139.75]	0.293
Albumin, g/L		39.60 [36.60, 42.42]	40.30 [37.20, 42.80]	38.15 [35.60, 41.40]	0.001	38.90 [36.30, 41.80]	39.80 [37.20, 42.50]	38.15 [35.60, 41.40]	0.021
Serum creatinine,μmol/L		89.95 [73.62, 108.98]	87.25 [73.10, 103.08]	95.85 [78.68, 127.12]	0.001	92.00 [75.10, 117.60]	88.90 [73.85, 99.40]	95.85 [78.68, 127.12]	0.010
Low density lipoprotein, mmol/L		2.03 [1.57, 2.55]	2.04 [1.57, 2.56]	2.02 [1.56, 2.50]	0.935	2.02 [1.53, 2.57]	2.01 [1.52, 2.62]	2.02 [1.56, 2.50]	0.794
Triglyceride, mmol/L		0.98 [0.74, 1.30]	0.98 [0.74, 1.38]	0.93 [0.73, 1.16]	0.094	0.88 [0.69, 1.16]	0.83 [0.67, 1.15]	0.93 [0.73, 1.16]	0.364
Preoperative IABP (%)		8 (1.9)	4 (1.3)	4 (4.1)	0.174	6 (3.2)	2 (2.3)	4 (4.1)	0.789
Preoperative ECMO (%)		6 (1.4)	1 (0.3)	5 (5.1)	0.003	6 (3.2)	1 (1.1)	5 (5.1)	0.272
Smoking history (%)		167 (40.1)	135 (42.5)	32 (32.7)	0.107	52 (28.1)	20 (23.0)	32 (32.7)	0.195
Cardiac surgery history (%)		114 (27.4)	72 (22.6)	42 (42.9)	<0.001	64 (34.6)	22 (25.3)	42 (42.9)	0.019
Charlson comorbidity index (%)					0.033				0.698
	0	229 (55.0)	180 (56.6)	49 (50.0)		98 (53.0)	49 (56.3)	49 (50.0)	
	1	120 (28.8)	95 (29.9)	25 (25.5)		47 (25.4)	22 (25.3)	25 (25.5)	
	2	53 (12.7)	36 (11.3)	17 (17.3)		31 (16.8)	14 (16.1)	17 (17.3)	
	≥3	14 (3.4)	7 (2.2)	7 (7.1)		9 (4.9)	2 (2.2)	7 (7.1)	
Preoperative hypertension (%)		71 (17.1)	60 (18.9)	11 (11.2)	0.109	19 (10.3)	8 (9.2)	11 (11.2)	0.833
Preoperative diabetes (%)		60 (14.4)	44 (13.8)	16 (16.3)	0.653	28 (15.1)	12 (13.8)	16 (16.3)	0.784
Waiting time		27.00 [20.00, 36.00]	27.50 [20.25, 35.00]	26.00 [19.00, 36.00]	0.686	26.00 [19.00, 36.00]	26.00 [20.00, 35.00]	26.00 [19.00, 36.00]	0.857
**Donor variables**									
Donor age		38.00 [26.00, 46.00]	38.00 [26.00, 46.00]	38.00 [26.00, 45.00]	0.799	38.00 [25.00, 45.00]	36.00 [25.00, 44.00]	38.00 [26.00, 45.00]	0.583
Donor gender (%)					0.418				0.352
	Male	366 (88.0)	277 (87.1)	89 (90.8)		172 (93.0)	83 (95.4)	89 (90.8)	
	Female	50 (12.0)	41 (12.9)	9 (9.2)		13 (7.0)	4 (4.6)	9 (9.2)	
Donor cold ischemic time		354.00 [302.75, 400.00]	353.00 [303.00, 398.25]	356.50 [302.00, 400.00]	0.676	362.00 [302.00, 409.00]	366.00 [304.00, 429.50]	356.50 [302.00, 400.00]	0.451
Donor/recipient BMI		0.97 [0.86, 1.13]	0.97 [0.86, 1.12]	0.99 [0.89, 1.20]	0.059	1.01 [0.90, 1.18]	1.02 [0.90, 1.13]	0.99 [0.89, 1.20]	0.854
**Platelet**								
*n*		416.000	353.000	63.000		122.000	59.000	63.000	
**Recipient preoperative variable**									
Gender (%)					0.058				1.000
	Male, *n*	325 (78.1)	282 (79.9)	43 (68.3)		84 (68.9)	41 (69.5)	43 (68.3)	
	Female, *n*	91 (21.9)	71 (20.1)	20 (31.7)		38 (31.1)	18 (30.5)	20 (31.7)	
Age, y		50.00 [39.00, 57.00]	50.00 [39.00, 57.00]	53.00 [36.00, 58.50]	0.683	51.50 [38.00, 58.00]	51.00 [39.00, 56.50]	53.00 [36.00, 58.50]	0.739
BMI, kg/m^2^		22.84 [19.90, 25.36]	22.77 [20.07, 25.46]	23.38 [19.43, 25.03]	0.610	22.68 [19.75, 24.65]	22.49 [20.92, 24.23]	23.38 [19.43, 25.03]	0.814
Blood type (%)					0.092				0.136
	A	138 (33.2)	117 (33.1)	21 (33.3)		45 (36.9)	24 (40.7)	21 (33.3)	
	B	116 (27.9)	91 (25.8)	25 (39.7)		37 (30.3)	12 (20.3)	25 (39.7)	
	O	132 (31.7)	118 (33.4)	14 (22.2)		33 (27.0)	19 (32.2)	14 (22.2)	
	AB	30 (7.2)	27 (7.6)	3 (4.8)		7 (5.7)	4 (6.8)	3 (4.8)	
Red blood cell count 10^∧^12/L		4.44 [4.00, 4.83]	4.47 [4.03, 4.86]	4.27 [3.89, 4.72]	0.037	4.31 [3.92, 4.72]	4.35 [3.96, 4.72]	4.27 [3.89, 4.72]	0.494
Platelet count, 10^∧^9/L		171.00 [134.75, 218.25]	173.00 [136.00, 221.00]	160.00 [128.50, 201.00]	0.065	166.00 [137.50, 208.75]	170.00 [146.00, 211.00]	160.00 [128.50, 201.00]	0.164
White blood cell count, 10^∧^9/L		6.38 [4.95, 7.99]	6.34 [5.09, 7.98]	6.45 [4.35, 8.18]	0.353	6.38 [4.65, 7.91]	6.04 [4.95, 7.84]	6.45 [4.35, 8.18]	0.500
Hemoglobin, g/L		136.50 [119.75, 147.00]	137.00 [121.00, 147.00]	133.00 [113.50, 142.50]	0.085	133.50 [115.00, 141.75]	134.00 [116.50, 140.00]	133.00 [113.50, 142.50]	0.824
Albumin, g/L		39.60 [36.60, 42.42]	40.10 [37.20, 42.60]	37.70 [34.10, 41.10]	0.001	38.70 [35.52, 41.88]	39.60 [37.30, 42.35]	37.70 [34.10, 41.10]	0.015
Serum creatinine,μmol/L		89.95 [73.62, 108.98]	88.60 [72.80, 105.30]	97.60 [78.95, 122.40]	0.009	92.70 [78.50, 115.07]	90.00 [77.45, 99.95]	97.60 [78.95, 122.40]	0.087
Low density lipoprotein, mmol/L		2.03 [1.57, 2.55]	2.05 [1.57, 2.59]	2.01 [1.65, 2.40]	0.517	1.99 [1.46, 2.45]	1.83 [1.45, 2.49]	2.01 [1.65, 2.40]	0.497
Triglyceride, mmol/L		0.98 [0.74, 1.30]	0.99 [0.75, 1.37]	0.81 [0.70, 1.14]	0.053	0.81 [0.66, 1.13]	0.81 [0.64, 1.12]	0.81 [0.70, 1.14]	0.494
Preoperative IABP (%)		8 (1.9)	6 (1.7)	2 (3.2)	0.774	2 (1.6)	0 (0.0)	2 (3.2)	0.505
Preoperative ECMO (%)		6 (1.4)	5 (1.4)	1 (1.6)	1.000	1 (0.8)	0 (0.0)	1 (1.6)	1.000
Smoking history (%)		167 (40.1)	155 (43.9)	12 (19.0)	<0.001	21 (17.2)	9 (15.3)	12 (19.0)	0.753
Cardiac surgery (%)		114 (27.4)	94 (26.6)	20 (31.7)	0.493	40 (32.8)	20 (33.9)	20 (31.7)	0.952
Charlson comorbidity index (%)					0.760				0.584
	0	229 (55.0)	195 (55.2)	34 (54.0)		73 (59.8)	39 (66.1)	34 (54.0)	
	1	120 (28.8)	104 (29.5)	16 (25.4)		28 (23.0)	12 (20.3)	16 (25.4)	
	2	53 (12.7)	42 (11.9)	11 (17.5)		18 (14.8)	7 (11.9)	11 (17.5)	
	≥3	14 (3.4)	12 (3.5)	2 (3.2)		3 (2.5)	1 (1.7)	2 (3.2)	
Preoperative hypertension (%)		71 (17.1)	64 (18.1)	7 (11.1)	0.237	13 (10.7)	6 (10.2)	7 (11.1)	1.000
Preoperative diabetes (%)		60 (14.4)	51 (14.4)	9 (14.3)	1.000	18 (14.8)	9 (15.3)	9 (14.3)	1.000
Waiting time		27.00 [20.00, 36.00]	27.00 [20.00, 36.00]	26.00 [21.00, 35.00]	0.766	27.00 [21.00, 37.00]	28.00 [20.50, 39.50]	26.00 [21.00, 35.00]	0.494
**Donor variables**									
Donor age		38.00 [26.00, 46.00]	37.00 [26.00, 46.00]	40.00 [22.50, 44.50]	0.765	39.50 [23.25, 45.75]	39.00 [24.00, 46.50]	40.00 [22.50, 44.50]	0.614
Donor gender (%)					1.000				0.884
	Male	366 (88.0)	311 (88.1)	55 (87.3)		105 (86.1)	50 (84.7)	55 (87.3)	
	Female	50 (12.0)	42 (11.9)	8 (12.7)		17 (13.9)	9 (15.3)	8 (12.7)	
Donor cold ischemic time		354.00 [302.75, 400.00]	350.00 [302.00, 400.00]	366.00 [313.50, 400.00]	0.284	375.00 [308.50, 436.50]	390.00 [305.50, 459.00]	366.00 [313.50, 400.00]	0.148
Donor/recipient BMI		0.97 [0.86, 1.13]	0.98 [0.86, 1.13]	0.96 [0.85, 1.13]	0.598	0.96 [0.86, 1.12]	0.96 [0.86, 1.12]	0.96 [0.85, 1.13]	0.890
**Plasma**							
*n*		416.000	378.000	38.000		72.000	34.000	38.000	
**Recipient preoperative variable**									
Gender (%)					0.189				1.000
	Male, *n*	325 (78.1)	299 (79.1)	26 (68.4)		50 (69.4)	24 (70.6)	26 (68.4)	
	Female, *n*	91 (21.9)	79 (20.9)	12 (31.6)		22 (30.6)	10 (29.4)	12 (31.6)	
Age, y		50.00 [39.00, 57.00]	50.50 [39.25, 57.00]	48.00 [34.25, 57.75]	0.547	46.00 [32.25, 56.00]	41.00 [28.25, 53.00]	48.00 [34.25, 57.75]	0.097
BMI, kg/m^2^		22.84 [19.90, 25.36]	22.84 [19.86, 25.49]	22.75 [20.29, 24.24]	0.653	22.15 [20.71, 24.23]	22.00 [21.05, 24.17]	22.75 [20.29, 24.24]	0.748
Blood type (%)					0.896				0.515
	A	138 (33.2)	124 (32.8)	14 (36.8)		22 (30.6)	8 (23.5)	14 (36.8)	
	B	116 (27.9)	105 (27.8)	11 (28.9)		20 (27.8)	9 (26.5)	11 (28.9)	
	O	132 (31.7)	122 (32.3)	10 (26.3)		24 (33.3)	14 (41.2)	10 (26.3)	
	AB	30 (7.2)	27 (7.1)	3 (7.9)		6 (8.3)	3 (8.8)	3 (7.9)	
Red blood cell count 10^∧^12/L		4.44 [4.00, 4.83]	4.47 [4.01, 4.85]	4.26 [3.67, 4.60]	0.028	4.31 [3.92, 4.72]	4.55 [3.95, 4.78]	4.26 [3.67, 4.60]	0.141
Platelet count, 10^∧^9/L		171.00 [134.75, 218.25]	171.50 [136.25, 220.00]	161.00 [117.50, 199.75]	0.113	161.00 [128.25, 197.25]	161.00 [141.75, 193.00]	161.00 [117.50, 199.75]	0.826
White blood cell count, 10^∧^9/L		6.38 [4.95, 7.99]	6.33 [5.00, 7.93]	6.74 [4.45, 8.96]	0.605	6.60 [4.48, 9.00]	6.54 [4.96, 8.85]	6.74 [4.45, 8.96]	0.937
Hemoglobin, g/L		136.50 [119.75, 147.00]	137.00 [121.00, 147.00]	134.00 [112.50, 141.75]	0.177	135.00 [116.75, 142.00]	135.00 [123.50, 141.50]	134.00 [112.50, 141.75]	0.423
Albumin, g/L		39.60 [36.60, 42.42]	40.15 [37.20, 42.50]	36.10 [33.05, 39.98]	<0.001	38.70 [35.12, 41.95]	40.85 [38.12, 42.50]	36.10 [33.05, 39.98]	<0.001
Serum creatinine,μmol/L		89.95 [73.62, 108.98]	89.25 [73.78, 106.62]	93.40 [74.20, 138.52]	0.087	92.60 [72.52, 124.48]	91.60 [73.75, 101.27]	93.40 [74.20, 138.52]	0.310
Low density lipoprotein, mmol/L		2.03 [1.57, 2.55]	2.05 [1.57, 2.56]	1.92 [1.49, 2.43]	0.275	1.85 [1.47, 2.26]	1.79 [1.46, 2.19]	1.92 [1.49, 2.43]	0.604
Triglyceride, mmol/L		0.98 [0.74, 1.30]	0.98 [0.74, 1.37]	0.82 [0.72, 1.11]	0.046	0.81 [0.64, 1.10]	0.74 [0.62, 1.08]	0.82 [0.72, 1.11]	0.423
Preoperative IABP (%)		8 (1.9)	5 (1.3)	3 (7.9)	0.028	4 (5.6)	1 (2.9)	3 (7.9)	0.689
Preoperative ECMO (%)		6 (1.4)	5 (1.3)	1 (2.6)	1.000	1 (1.4)	0 (0.0)	1 (2.6)	1.000
Smoking history (%)		167 (40.1)	160 (42.3)	7 (18.4)	0.007	14 (19.4)	7 (20.6)	7 (18.4)	1.000
Cardiac surgery (%)		114 (27.4)	99 (26.2)	15 (39.5)	0.119	22 (30.6)	7 (20.6)	15 (39.5)	0.139
Charlson comorbidity index (%)					0.515				0.209
	0	229 (55.0)	211 (55.8)	18 (47.4)		41 (56.9)	23 (67.6)	18 (47.4)	
	1	120 (28.8)	109 (28.8)	11 (28.9)		18 (25.0)	7 (20.6)	11 (28.9)	
	2	53 (12.7)	46 (12.2)	7 (18.4)		9 (12.5)	2 (5.9)	7 (18.4)	
	≥3	14 (3.4)	12 (3.1)	2 (5.3)		4 (5.6)	2 (5.8)	2 (5.3)	
Preoperative hypertension (%)		71 (17.1)	65 (17.2)	6 (15.8)	1.000	10 (13.9)	4 (11.8)	6 (15.8)	0.879
Preoperative diabetes (%)		60 (14.4)	54 (14.3)	6 (15.8)	0.993	10 (13.9)	4 (11.8)	6 (15.8)	0.879
Waiting time		27.00 [20.00, 36.00]	27.00 [20.00, 35.00]	32.00 [21.25, 38.75]	0.265	32.00 [24.00, 40.00]	34.00 [26.25, 40.00]	32.00 [21.25, 38.75]	0.196
**Donor variables**									
Donor age		38.00 [26.00, 46.00]	38.00 [26.00, 46.00]	38.00 [26.00, 45.00]	0.559	40.50 [27.50, 47.00]	41.50 [30.00, 47.00]	38.00 [26.00, 45.00]	0.615
Donor gender (%)					1.000				1.000
	Male	366 (88.0)	333 (88.1)	33 (86.8)		62 (86.1)	29 (85.3)	33 (86.8)	
	Female	50 (12.0)	45 (11.9)	5 (13.2)		10 (13.9)	5 (14.7)	5 (13.2)	
Donor cold ischemic time		354.00 [302.75, 400.00]	353.50 [303.25, 400.00]	360.00 [223.75, 399.75]	0.762	361.00 [159.50, 428.25]	374.50 [155.75, 478.50]	360.00 [223.75, 399.75]	0.407
Donor/recipient BMI		0.97 [0.86, 1.13]	0.97 [0.86, 1.13]	0.97 [0.89, 1.17]	0.545	0.97 [0.89, 1.13]	0.98 [0.88, 1.12]	0.97 [0.89, 1.17]	0.676

*INR, International Normalized Ratio; IABP, Intraaortic Balloon Counterpulsation; ECMO, extracorporeal membrane oxygenation; BMI, Body Mass Index*.

*The p-values were calculated by Chi-square test or Fisher's precision probability test for continuous variables, and Mann-Whitney U rank-sum test for categorical variables, as appropriate*.

Moreover, a high amount of RBC transfusion was significantly related to the high ratio of preoperative ECMO application (*p* = 0.003), high ratio of cardiac surgery history (*p* < 0.001), and high CCI (*p* = 0.033). A high amount of platelet transfusion was significantly related to the low ratio of smoking history (*p* < 0.001). A high amount of plasma transfusion was related to a high ratio of preoperative IABP application (*p* = 0.028), and a low ratio of smoking history (*p* = 0.007).

### Propensity Score Matching and Survival Analysis

**Figure 3:**
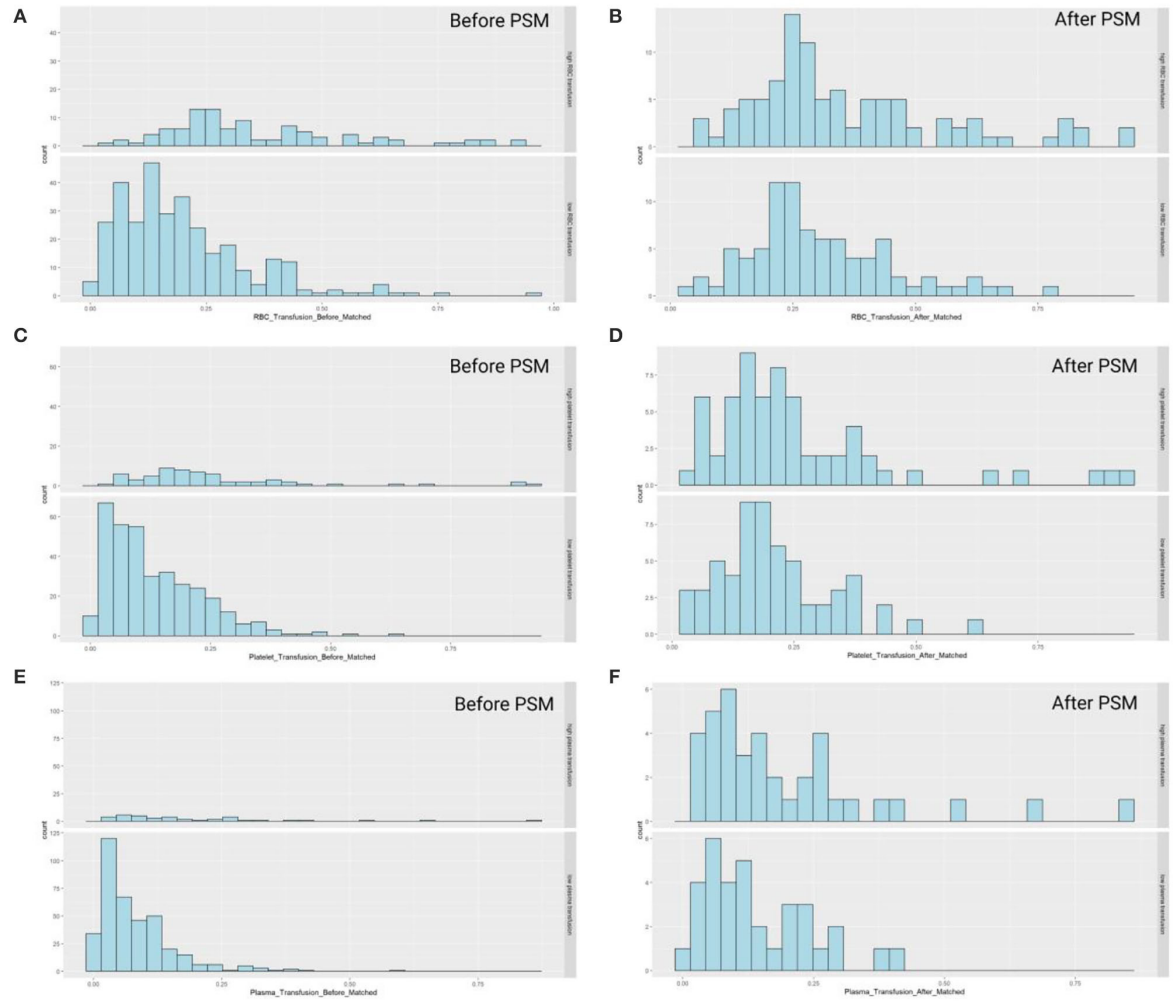
The scores of three components presented skewed distribution prior to the propensity score matching, and approximately normally distributed after match. The score distributions were separately shown as below, in which the study population was divided into two groups based on the transfusion amount of: **(A)** red blood cell (before matching); **(B)** red blood cell (after matching); **(C)** platelet (before matching); **(D)** platelet (after matching); **(E)** plasma (before matching); **(F)** plasma (after matching).

The propensity score matching was used for analysis in this study with the caliper of 0.02 and no replacement. All baseline characteristics, including recipient variables, personal history, and donor variables, were considered in matching. The distribution of propensity scores before and after matching was shown in Figure 3. Unmatched recipients score tended to locate in a right-skewed distribution, which turned into a balanced one after propensity score matching. Most of the baseline characteristics posed no statistical significance after matching, showing that the bias in baseline indicators achieved a good balance and contributed to the two groups equally.

**Figure 4:**
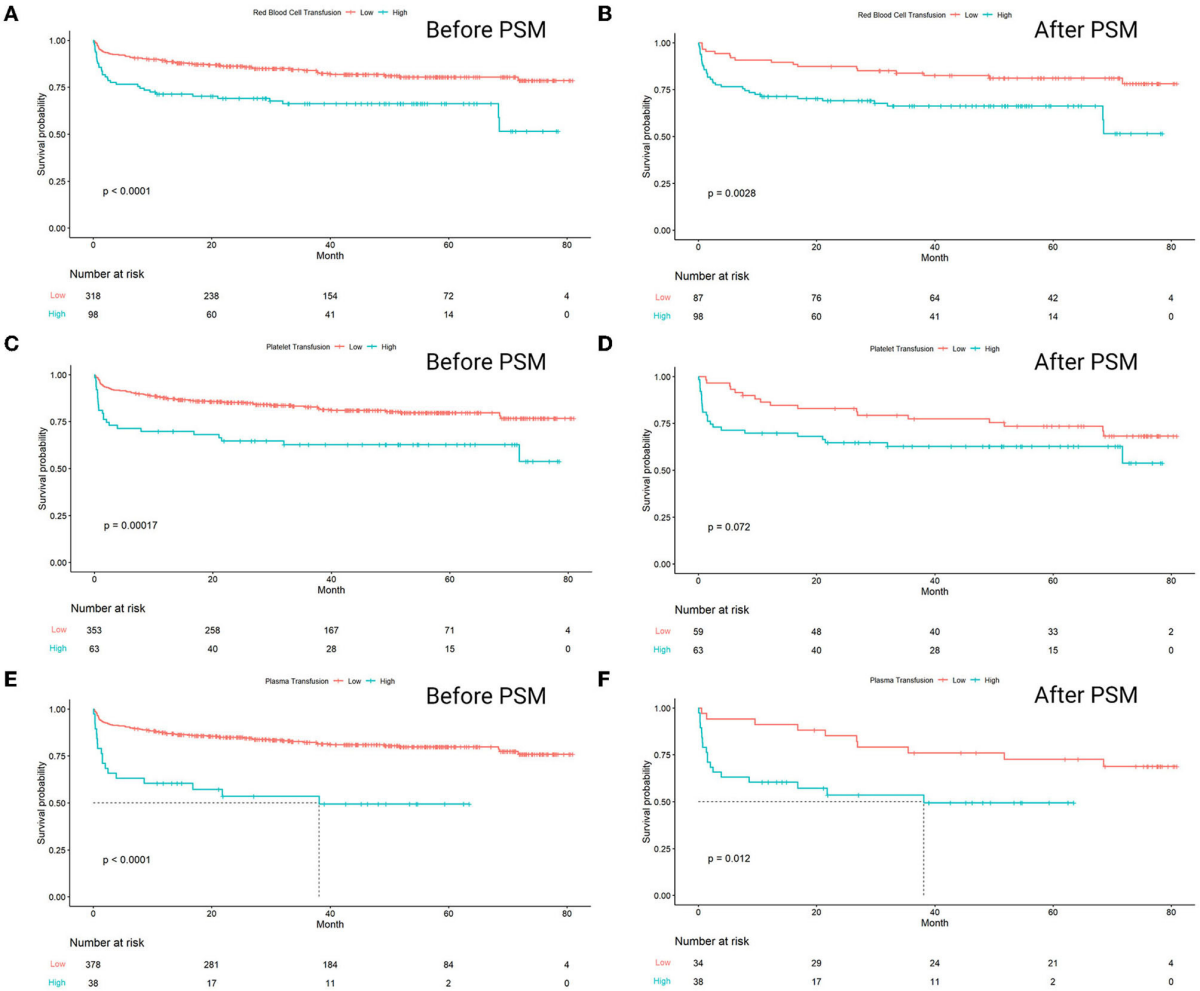
Kaplan–Meier analysis estimates survival after heart transplantation with log-rank test, comparing the outcomes between high amount transfusion group and low amount transfusion group. The analysis results were separately shown below, in which the study population was divided into two groups based on the transfusion amount of **(A)** Red blood cell (beforematching), *p* < 0.0001; **(B)** red blood cell (aftermatching), *p* = 0.0028; **(C)** platelet (beforematching), *p* = 0.00017; **(D)** platelet (aftermatching), *p* = 0.072; **(E)** plasma (beforematching), *p* < 0.0001; **(F)** plasma (aftermatching), *p* = 0.012.

The result of Kaplan–Meier survival analysis showed that the survival curves of three pairs of groups were all significantly separated. The groups with high-amounts transfusion of RBC, platelet, and plasma had significantly lower survival rates compared to the low-amount ones (*p* < 0.0001, *p* = 0.00017, *p* < 0.0001, respectively). The significant separation of the groups with a higher amount of RBC and plasma transfusion remained consistent after propensity score matching, with *p* = 0.0028 and *p* = 0.012, respectively. However, the survival results failed to expose significant separation in a high amount of platelet transfusion group, with *p* = 0.072 (Figure 4).

### Postoperative Events and Mortality

Table 2 presented the outcomes after transplantation. All patients were followed up for more than 1 year after the operation. Early postoperative events and mortality were retrieved from the electronic medical record system.

**Table 2:** Postoperative outcome of the 416 patients in the study cohorts.

**Variables**	**Before matched**				**After matched**			
	**Overall**	**Low amount≤(9 unit)**	**High amount (>9 unit)**	***p***	**Overall**	**Low amount≤(9 unit)**	**High amount (>9 unit)**	***p***
**Red blood cell**							
*n*	416	318	98		185	87	98	
**Early postoperative events**							
Postoperative CRRT (%)	59 (14.2)	26 (8.2)	33 (33.7)	<0.001	39 (21.1)	6 (6.9)	33 (33.7)	<0.001
Postoperative IABP (%)	165 (39.7)	113 (35.5)	52 (53.1)	0.003	76 (41.1)	24 (27.6)	52 (53.1)	0.001
Postoperative ECMO (%)	24 (5.8)	5 (1.6)	19 (19.4)	<0.001	20 (10.8)	1 (1.1)	19 (19.4)	<0.001
Respiratory complications (%)	254 (61.1)	185 (58.2)	69 (70.4)	0.04	116 (62.7)	47 (54.0)	69 (70.4)	0.032
Neurological complications (%)	29 (7.0)	17 (5.3)	12 (12.2)	0.034	17 (9.2)	5 (5.7)	12 (12.2)	0.203
Renal complications (%)	68 (16.3)	41 (12.9)	27 (27.6)	0.001	32 (17.3)	5 (5.7)	27 (27.6)	<0.001
Liver damage (%)	32 (7.7)	15 (4.7)	17 (17.3)	<0.001	20 (10.8)	3 (3.4)	17 (17.3)	0.005
Septic shock (%)	11 (2.6)	6 (1.9)	5 (5.1)	0.169	8 (4.3)	3 (3.4)	5 (5.1)	0.849
Secondary thoracotomy (%)	17 (4.1)	2 (0.6)	15 (15.3)	<0.001	15 (8.1)	0 (0.0)	15 (15.3)	<0.001
ICU stay time (day)	219.75 [162.75, 290.75]	211.50 [159.25, 274.75]	249.50 [192.00, 407.75]	<0.001	235.00 [186.00, 325.00]	227.00 [174.50, 277.50]	249.50 [192.00, 407.75]	0.023
**Mortality variables**								
Peritransplant period mortality (%)	29 (7.0)	15 (4.7)	14 (14.3)	0.002	17 (9.2)	3 (3.4)	14 (14.3)	0.022
1-year mortality	76 (18.3)	43 (13.5)	33 (33.7)	<0.001	41 (22.2)	8 (9.2)	33 (33.7)	<0.001
5-year mortality	86 (20.7)	54 (17.0)	32 (32.7)	0.001	48 (25.9)	16 (18.4)	32 (32.7)	0.041
**Platelet**							
*n*	416	353	63		122	59	63	
**Early postoperative events**							
Postoperative CRRT (%)	59 (14.2)	33 (9.3)	26 (41.3)	<0.001	29 (23.8)	3 (5.1)	26 (41.3)	<0.001
Postoperative IABP (%)	165 (39.7)	123 (34.8)	42 (66.7)	<0.001	63 (51.6)	21 (35.6)	42 (66.7)	0.001
Postoperative ECMO (%)	24 (5.8)	8 (2.3)	16 (25.4)	<0.001	16 (13.1)	0 (0.0)	16 (25.4)	<0.001
Respiratory complications (%)	254 (61.1)	206 (58.4)	48 (76.2)	0.011	81 (66.4)	33 (55.9)	48 (76.2)	0.03
Neurological complications (%)	29 (7.0)	21 (5.9)	8 (12.7)	0.095	13 (10.7)	5 (8.5)	8 (12.7)	0.644
Renal complications (%)	68 (16.3)	46 (13.0)	22 (34.9)	<0.001	24 (19.7)	2 (3.4)	22 (34.9)	<0.001
Liver damage (%)	32 (7.7)	18 (5.1)	14 (22.2)	<0.001	17 (13.9)	3 (5.1)	14 (22.2)	0.014
Septic shock (%)	11 (2.6)	8 (2.3)	3 (4.8)	0.477	4 (3.3)	1 (1.7)	3 (4.8)	0.659
Secondary thoracotomy (%)	17 (4.1)	7 (2.0)	10 (15.9)	<0.001	11 (9.0)	1 (1.7)	10 (15.9)	0.016
ICU stay time (day)	219.75 [162.75, 290.75]	210.00 [157.00, 270.00]	336.00 [239.75, 438.50]	<0.001	269.50 [213.25, 354.75]	235.00 [202.75, 281.50]	336.00 [239.75, 438.50]	<0.001
**Mortality variables**								
Peritransplant period mortality (%)	29 (7.0)	17 (4.8)	12 (19.0)	<0.001	12 (9.8)	0 (0.0)	12 (19.0)	0.001
1-year mortality	76 (18.3)	56 (15.9)	20 (31.7)	0.005	29 (23.8)	9 (15.3)	20 (31.7)	0.054
5-year mortality	86 (20.7)	63 (17.8)	23 (36.5)	0.001	38 (31.1)	15 (25.4)	23 (36.5)	0.26
**Plasma**							
*n*	416	378	38		72	34	38	
**Early postoperative events**							
Postoperative CRRT (%)	59 (14.2)	33 (8.7)	26 (68.4)	<0.001	29 (40.3)	3 (8.8)	26 (68.4)	<0.001
Postoperative IABP (%)	165 (39.7)	138 (36.5)	27 (71.1)	<0.001	43 (59.7)	16 (47.1)	27 (71.1)	0.067
Postoperative ECMO (%)	24 (5.8)	10 (2.6)	14 (36.8)	<0.001	17 (23.6)	3 (8.8)	14 (36.8)	0.012
Respiratory complications (%)	254 (61.1)	223 (59.0)	31 (81.6)	0.011	50 (69.4)	19 (55.9)	31 (81.6)	0.035
Neurological complications (%)	29 (7.0)	22 (5.8)	7 (18.4)	0.01	9 (12.5)	2 (5.9)	7 (18.4)	0.212
Renal complications (%)	68 (16.3)	50 (13.2)	18 (47.4)	<0.001	20 (27.8)	2 (5.9)	18 (47.4)	<0.001
Liver damage (%)	32 (7.7)	20 (5.3)	12 (31.6)	<0.001	15 (20.8)	3 (8.8)	12 (31.6)	0.037
Septic shock (%)	11 (2.6)	8 (2.1)	3 (7.9)	0.113	3 (4.2)	0 (0.0)	3 (7.9)	0.279
Secondary thoracotomy (%)	17 (4.1)	8 (2.1)	9 (23.7)	<0.001	10 (13.9)	1 (2.9)	9 (23.7)	0.028
ICU stay time (day)	219.75 [162.75, 290.75]	213.50 [159.00, 273.50]	418.00 [305.25, 745.50]	<0.001	310.50 [233.25, 452.25]	263.50 [220.25, 312.25]	418.00 [305.25, 745.50]	<0.001
**Mortality variables**								
Peritransplant period mortality (%)	29 (7.0)	21 (5.6)	8 (21.1)	0.001	9 (12.5)	1 (2.9)	8 (21.1)	0.05
1-year mortality	76 (18.3)	59 (15.6)	17 (44.7)	<0.001	20 (27.8)	3 (8.8)	17 (44.7)	0.002
5-year mortality	86 (20.7)	68 (18.0)	18 (47.4)	<0.001	27 (37.5)	9 (26.5)	18 (47.4)	0.113

*INR, International Normalized Ratio; IABP, Intraaortic Balloon Counterpulsation; ECMO, extracorporeal membrane oxygenation; BMI, Body Mass Index*.

*The p-values were calculated by Chi-square test or Fisher's precision probability test for continuous variables, and Mann-Whitney U rank-sum test for categorical variables, as appropriate*.

The high-amount RBC transfusion cohort had higher risk of respiratory complications (*p* = 0.032), renal complications (*p* < 0.001), liver damage (*p* = 0.005), postoperative IABP use (*p* = 0.001), postoperative ECMO use (*p* < 0.001), postoperative CRRT use (*p* < 0.001), secondary thoracotomy (*p* < 0.001), and longer ICU stay time (*p* <0.023) in the short-term period after operation. The high-amount platelet transfusion cohort had higher risk of respiratory complications (*p* = 0.03), renal complications (*p* < 0.001), liver damage (*p* = 0.014), postoperative IABP use (*p* = 0.001), postoperative ECMO use (*p* < 0.001), postoperative CRRT use (*p* < 0.001), secondary thoracotomy (*p* = 0.016), and longer ICU stay time (*p* < 0.001). The high-amount plasma transfusion cohort had higher risk of respiratory complications (*p* = 0.035), renal complications (*p* < 0.001), liver damage (*p* = 0.037), postoperative ECMO use (*p* = 0.012), postoperative CRRT use (*p* < 0.001), secondary thoracotomy (*p* = 0.028), and longer ICU stay time (*p* < 0.001).

The mortality variables revealed that the high-amount RBC transfusion cohort had a higher risk of peritransplant period mortality (*p* = 0.002), 1-year mortality (*p* < 0.001), and 5-year mortality (*p* = 0.041). The high-amount platelet transfusion cohort had a higher risk of peritransplant period mortality (*p* = 0.001). The high-amount plasma transfusion cohort had a higher risk of peritransplant period mortality (*p* = 0.05), and 1-year mortality (*p* = 0.002).

### Univariate and Multivariate Cox Analysis

Risk factors for overall mortality after HTx were tested in univariate and multivariate Cox proportional hazards regression model. The result of univariate analysis showed that the risk factors of mortality were neurological complications (HR = 4.290, 95% CI 2.485–7.407, *p* < 0.001), liver damage (HR = 4.170, 95% CI 2.416–7.198, *p* < 0.001), respiratory complications (HR = 2.296, 95% CI 1.416–3.722, *p* = 0.001), postoperative CRRT use (HR = 3.709, 95% CI 2.301–5.979, *p* < 0.001), postoperative IABP use (HR = 1.906, 95% CI 1.257–2.891, *p* = 0.002), renal complications (HR = 3.789, 95% CI 2.371–6.054, *p* < 0.001), septic shock (HR = 3.014, 95% CI 1.219–7.451, *p* = 0.017), postoperative ECMO use (HR = 3.156, 95% CI 1.631–6.107, *p* = 0.001), and secondary thoracotomy (HR = 3.441, 95% CI 1.660–7.133, *p* = 0.001).

Multivariate analysis showed that neurological complications (HR = 2.913, 95% CI 1.543–5.501, *p* = 0.001), liver damage (HR = 2.336, 95% CI 1.216–4.485, *p* = 0.011), respiratory complications (HR = 1.684, 95% CI 1.014–2.798, *p* = 0.044) were independent and significant predictors of worse outcomes after transplantation (Table 3).

**Table 3:** Results of proportional hazards regression model.

**Postoperative events**	**Univariate**			**Multivariate**		
	**β**	**HR (95%CI)**	***p-value***	**β**	**HR (95%CI)**	***p-value***
Neurological complications	1.456	4.290 (2.485, 7.407)	0.000	1.069	2.913 (1.543, 5.501)	0.001
Liver damage	1.428	4.170 (2.416, 7.198)	0.000	0.848	2.336 (1.216, 4.485)	0.011
Respiratory complications	0.831	2.296 (1.416, 3.722)	0.001	0.521	1.684 (1.014, 2.798)	0.044
Postoperative CRRT	1.311	3.709 (2.301, 5.979)	0.000	0.607	1.835 (0.991, 3.398)	0.054
Postoperative IABP	0.645	1.906 (1.257, 2.891)	0.002	0.439	1.551 (0.992, 2.423)	0.054
Renal complications	1.332	3.789 (2.371, 6.054)	0.000	0.532	1.702 (0.906, 3.198)	0.098
Septic shock	1.103	3.014 (1.219, 7.451)	0.017	−0.738	0.478 (0.156, 1.462)	0.196
Postoperative ECMO	1.149	3.156 (1.631, 6.107)	0.001	−0.396	0.673 (0.254, 1.786)	0.427
Secondary thoracotomy	1.236	3.441 (1.660, 7.133)	0.001	0.291	1.338 (0.523, 3.422)	0.544

### Combining the Transfusion of RBC, Platelet, and Plasma in the Prediction of Peritransplant Period Survival

**Figure 5:**
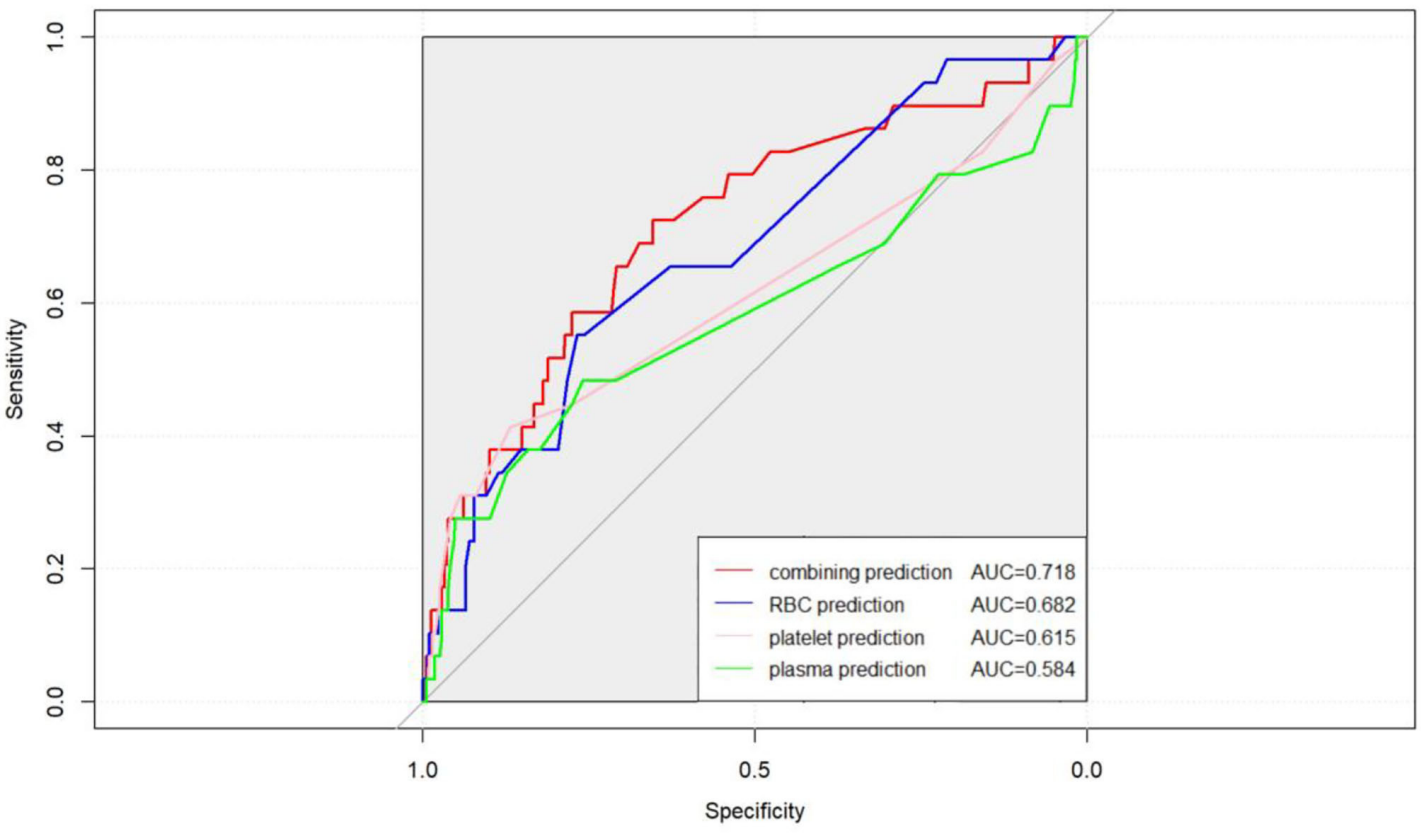
Combining the transfusion amount of RBC, platelet, and plasma together had a better accuracy of peritransplant period prognostic assessment to heart transplantation (AUC = 0.718 *vs*. AUC = 0.682 for RBC, AUC = 0.615 for platelet, and AUC = 0.584 for plasma).

In the last part of the analysis, we explored the predictive value of the RBC, platelet, and plasma transfusion amount in short-term survival. A C-index test was conducted to analyze the data before and after propensity score matching. The combining predictions of RBC, platelet, and plasma transfusion were considered in the analysis compared to a single prediction. The results of the analysis were shown in Figure 5, in which the combining predictions posed a better predictive capability than the single prediction in peritransplant period survival outcomes in the whole population.

## Discussion

In consideration of the organ shortage and poorly predictive outcome after HTx, it was of great significance to explore and evaluate the risk factors that contribute to the prediction of prognosis. To our knowledge, the present study may be the largest-scale single-center cohort worldwide to separately explored whether high amounts of intraoperative transfusion of three major blood components were associated with clinical outcomes after HTx.

One of the major novel findings was that high-amount transfusions of RBC and plasma were associated with poor survival results. Patients with a higher amount of platelet transfusion had a greater proportion of unfavorable outcomes, although the survival difference failed to reach statistical significance (*p* = 0.085).

Furthermore, combining the transfusion amount of RBC, platelet, and plasma would be a considered predictive appliance for mortality in the peritransplant period. These findings would probably provide a recommendation about blood transfusion during HTx and instruct us to use blood more scientifically.

RBC transfusion is a common intraoperative intervention that maintains oxygen-carry capacity against surgical blood loss (5). It is not only an oxygen carrier but also applies hemoglobin as a central sensor of tissue hypoxia, transferring nitrite to nitric oxide to increase regional tissue blood flow (6, 7). However, the application of RBC transfusion could also lead to further physical impairment. The most threatening one is the potential of infectious agents and several allergic responses associated with RBC (8). Besides, improper transfusion volume could bring about transfusion-associated circulatory overload, leading to hypoxemia, respiratory distress, hypertension, and pulmonary edema (9). In the present study, the recipients with a higher amount of RBC transfusion had more potential for postoperative use of CRRT, ECMO, and IABP, greater risk of respiratory complications, renal complications, liver damage, and secondary thoracotomy. Among them, respiratory complications and liver damage had been proved to be independent risk factors for overall mortality. The high occurrence of them may be contributed to the transfusion volume overload and immune response of blood products. Confounding factors were eliminated by PSM analysis, and the clinical characteristics of the research subject were comparable. Our results also indicated the recipients with a lower amount of RBC transfusion might obtain better survival results, shorter ICU stay time, and less rate of postoperative complications than the higher amount transfusion group.

Prevalence of platelet dysfunction and thrombocytopenia have increased in patients with cardiac surgery due to the widespread application of potent antiplatelet agents (3). Transfusing platelet directly is an effective treatment for decreasing coagulation. One unit of platelet transfusion is suggested to raise the average count of platelet by 15–25 ×10^9^/L (10). According to EACTS/EACTA Guidelines on patient blood management for adult cardiac surgery published in 2017, patients with a platelet count below 50 × 10^9^/L or on antiplatelet therapy with bleeding complications are recommended to receive platelet transfusion (Class IIa) (10). However, several studies suggested that platelet transfusion may be associated with complications and adverse outcomes after cardiac surgery (11, 12). As far as we know, there are currently no clinical studies focusing on platelet transfusion and outcomes after HTx. In the present study, patients in the high-amount platelet transfusion group had lower platelet count before transplantation, despite not reaching significance (*p* = 0.065). It indicated that those patients may suffer from platelet hypofunction preoperatively, and thus have higher requirements of intraoperative platelet transfusion, under the risk of unfavorable coagulation function. For the surgical procedure, a high platelet transfusion amount would probably reflect higher complexity and risk of surgical procedure for transplantation. Moreover, patients with high-amount platelet transfusion also presented higher rates of application of assisting devices, and a sequence of postoperative complications, including respiratory and renal complications. Previous evidence revealed that the transfused platelet interacts with immune response in lung microcirculation, leading to neutrophil sequestration, endothelial damage, and capillary leak (12). More attention, postoperative care, and in-time intervention may contribute to the recovery and longer survival of patients with a high amount of platelet transfusion. The confounding factors were minimized by propensity score matching, making the results comparable and interpretable.

Plasma transfusion is also a common therapy for intraoperative bleeding in cardiac surgery. The rate of plasma transfusion in patients undergoing cardiac surgery reached over 20% in some studies (13, 14). The hemodilution and consumption lead to a reduction of coagulation factors during the operation, which may result in serious bleeding, and plasma transfusion effectively improved such a situation (15, 16). Despite the potential benefit, higher volumes of plasma transfusion have been reported to cause an inferior outcome and higher volume of RBC transfusion after cardiac surgery (4). Similarly, the direct correlation between the amount of plasma transfusion and the outcome of HTx is also unexplored. In the present study, patients with high-amount plasma transfusion also had lower red blood cell count, lower albumin, and lower triglycerides. Malnutrition is highly prevalent in patients hospitalized for heart failure. Besides, more plasma transfusion indicates more intraoperative bleeding and derangement of coagulation factors, reflecting a higher potential of surgery difficulty and then triggering worse outcomes. A previous study had revealed that the state of malnutrition was associated with a poor prognosis and increased mortality in heart failure patients (17). Serum albumin level is one of the major risk scores for estimating malnutrition, while triglycerides can also reflect the nutrient situation of heart failure patients before transplantation. Lower albumin and triglycerides levels hint at risk of malnutrition, and so the worse outcomes after transplantation (18, 19). From the prognosis analysis, the high-amount plasma transfusion cohort exhibited a significantly higher risk of postoperative complications, device assistance, and worse survival result.

Overall, high-amount transfusions of RBC, platelet, and plasma were demonstrated to have a positive correlation with unfavorable outcomes after HTx, although the transfusion of platelet poses no statistical difference in survival. Blood transfusion often includes the transfusion of more than one component. A total of 389 out of all 416 patients in the cohort received different amounts of three blood components transfusion. Comprehensively, considering the components of blood transfusion would obtain better predictive results of peritransplant period survival than solely considering a single component. In the present study, a combining indicator was established and a better predictive effect of peritransplant period survival than every single indicator was obtained (AUC = 0.718 *vs*. AUC = 0.682 for RBC, AUC = 0.615 for platelet, and AUC = 0.584 for plasma). Further studies are necessary to assess these findings and determine the reasons behind the difference in risk of mortality.

Our study expanded previous findings on the relationship between blood transfusion and the outcomes of HTx. These findings would contribute to distinguishing the patients with higher risks of worse outcomes before or during the surgery, and benefit the intra- and postoperative care for recipients to obtain better results. Nonetheless, further exploration of the underlying mechanism that how high-amount transfusion affects the outcome of HTx was warranted. Moreover, deeper understanding between platelet transfusion and survival after transplantation were still required to be explored in future studies.

## Limitations

There were several limitations in the present study: First, this was a single-center, retrospective study and it might have introduced selection bias when enrolling patients. Although evaluating transfusion amounts of RBC, platelet, and plasma was an effective method to predict peritransplant period survival after HTx, studies with larger sample sizes were required to verify this.

## Data Availability Statement

The data analyzed in this study is subject to the following licenses/restrictions: Some or all data, models, or code generated or used during the study are proprietary or confidential in nature and may only be provided with restrictions (e.g., anonymized data). (List items and restrictions). Requests to access these datasets should be directed to FL, lifei_union@hust.edu.cn.

## Ethics Statement

The studies involving human participants were reviewed and approved by Ethics Committee of Tongji Medical College of Huazhong University of Science and Technology. The patients/participants provided their written informed consent to participate in this study.

## Author Contributions

YZ and LX were in charge of collecting, analyzing data, and writing this manuscript. ZC, JT, YW, and YL contributed to the discussion and provided additional advice. SC, ND, and FL gave their valuable and professional suggestions and guidance in organizing and drafting this manuscript. All authors contributed to the article and approved the submitted version.

## Funding

This study was supported by the National Natural Science Foundation of China (81974034).

## Conflict of Interest

The authors declare that the research was conducted in the absence of any commercial or financial relationships that could be construed as a potential conflict of interest.

## Publisher's Note

All claims expressed in this article are solely those of the authors and do not necessarily represent those of their affiliated organizations, or those of the publisher, the editors and the reviewers. Any product that may be evaluated in this article, or claim that may be made by its manufacturer, is not guaranteed or endorsed by the publisher.
