# Corrigendum: The predictive role of intraoperative blood transfusion components in the prognosis of heart transplantation

**DOI:** 10.3389/fcvm.2022.952663

**Published:** 2022-08-18

**Authors:** Yidan Zheng, Li Xu, Ziwen Cai, Jingrong Tu, Yuqi Liu, Yixuan Wang, Si Chen, Nianguo Dong, Fei Li

**Affiliations:** ^1^Department of Cardiovascular Surgery, Union Hospital, Tongji Medical College, Huazhong University of Science and Technology, Wuhan, China; ^2^Key Laboratory of Organ Transplantation, Ministry of Education, NHC Key Laboratory of Organ Transplantation, Key Laboratory of Organ Transplantation, Chinese Academy of Medical Sciences, Wuhan, China

**Keywords:** heart transplantation, blood transfusion, red blood cell, platelet, plasma

In the published article, there was an error in affiliation **1**. Instead of “Department of Cardiovascular Surgery, Tongji Medical College, Union Hospital, Huazhong University of Science and Technology, Wuhan, China,” it should be “Department of Cardiovascular Surgery, Union Hospital, Tongji Medical College, Huazhong University of Science and Technology, Wuhan, China.”

There was a mistake in Figure 4 as published. The incorrect figure was uploaded. The corrected Figure 4 appears below.

**Figure 4:**
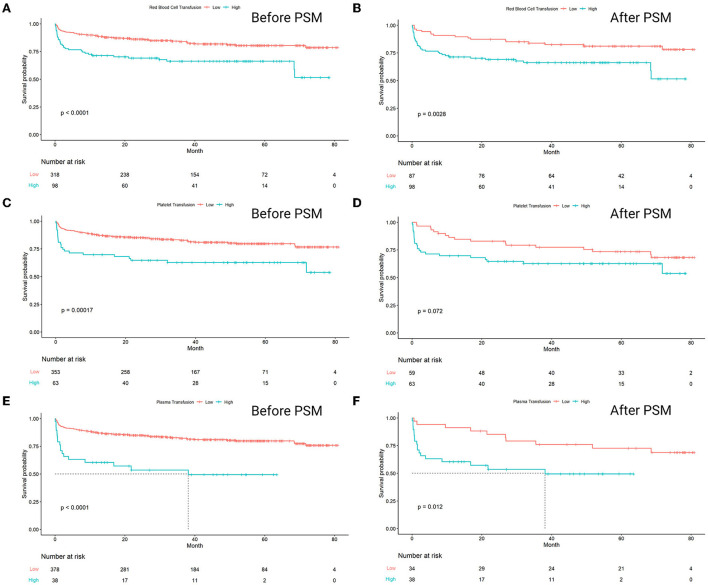
**(A)** Red blood cell (before matching), *p* < 0.0001; **(B)** red blood cell (after matching), *p* = 0.0028; **(C)** platelet (before matching), *p* = 0.00017; **(D)** platelet (after matching), *p* = 0.072; **(E)** plasma (before matching), *p* < 0.0001; **(F)** plasma (after matching), *p* = 0.012.

There was a mistake in the legend for Figure 4 as published. “(A) red blood cell (before matching), *p* < 0.0001; (B) red blood cell (after matching), *p* = 0.00073; (C) platelet (before matching), *p* = 0.00017; (D) platelet (after matching), *p* = 0.085; (E) plasma (before matching), *p* < 0.0001; (F) plasma (after matching), *p* = 0.018.” is incorrect.

The correct legend appears below.

“(A) Red blood cell (before matching), *p* < 0.0001; (B) red blood cell (after matching), *p* = 0.0028; (C) platelet (before matching), *p* = 0.00017; (D) platelet (after matching), *p* = 0.072; (E) plasma (before matching), *p* < 0.0001; (F) plasma (after matching), *p* = 0.012.”

There was an error. The data in Figure 4 was mis-described.

A correction has been made to “Result”, “Propensity Score Matching and Survival Analysis”, Paragraph 2:

“The result of Kaplan–Meier survival analysis showed that the survival curves of three pairs of groups were all significantly separated. The groups with high-amounts transfusion of RBC, platelet, and plasma had significantly lower survival rates compared to the low-amount ones (*p* < 0.0001, *p* = 0.00017, *p* < 0.0001, respectively). The significant separation of the groups with a higher amount of RBC and plasma transfusion remained consistent after propensity score matching, with *p* = 0.0028 and *p* = 0.012, respectively. However, the survival results failed to expose significant separation in a high amount of platelet transfusion group, with *p* = 0.072 (Figure 4).”

The authors apologize for this error and state that this does not change the scientific conclusions of the article in any way. The original article has been updated.

## Publisher's note

All claims expressed in this article are solely those of the authors and do not necessarily represent those of their affiliated organizations, or those of the publisher, the editors and the reviewers. Any product that may be evaluated in this article, or claim that may be made by its manufacturer, is not guaranteed or endorsed by the publisher.

